# Novel *N*-2-(Furyl)-2-(chlorobenzyloxyimino) Ethyl Piperazinyl Quinolones: Synthesis, Cytotoxic Evaluation and Structure-Activity Relationship

**Published:** 2015

**Authors:** Negar Mohammadhosseini, Mahboobeh Pordeli, Maliheh Safavi, Loghman Firoozpour, Fatame Amin, Sussan Kabudanian Ardestani, Najmeh Edraki, Abbas Shafiee, Alireza Foroumadi

**Affiliations:** a *Young Researchers and Elite Club, Islamshahr Branch, Islamic Azad University, Tehran, Iran.*; b *Institute of Biochemistry and Biophysics, University of Tehran, Tehran, Iran.*; c *Biotechnology Department, Iranian Research Organization for Science and Technology (IROST), Tehran, Iran. *; d *Drug Design and Development Research Center, Tehran University of Medical Sciences, Tehran, Iran.*; e *School of Chemistry, University College of Science, University of Tehran, Tehran, Iran.*; f *Medicinal and Natural Products Chemistry Research Center, Shiraz University of Medical Sciences, Shiraz, Iran. *; g *Department of Medicinal Chemistry, School of Pharmacy and Pharmaceutical Sciences Research Center, Tehran University of Medical Sciences, Tehran, Iran.*

**Keywords:** Furyl, *N*-pipearzinyl quinolones, Cytotoxic activity, Structure-activity Relationship

## Abstract

Quinolone antibacterials are one of the most important classes of pharmacological agents known as potent inhibitors of bacterial DNA gyrase and topoisomerase IV that efficiently inhibit DNA replication and transcription by generating several double-stranded DNA break. Some quinolone derivatives demonstrated inhibitory potential against eukaryote topoismarase II and substantial dose-dependent cytotoxic potential against some cancerous cells. In present study, synthesis and cytotoxic activity evaluation of new series of *N*-pipearzinyl quinolones containing *N*-2-(furyl-2 or 3-yl)-2-(chlorobenzyloxyimino) ethyl moiety 7a-i have been studied. Reaction of quinolone, with 2-bromo-1-(furan-2 or 3-yl)ethanone-*O*-substituted chlorobenzyloxime in DMF in presence of NaHCO_3_ at room temperature, gave the title compounds *N-*2-(furan-2 or 3-yl)-2-(chlorobenzyloxyiminoethyl) quinolone 7a-i. Synthesized compounds were further evaluated *in-vitro* against three human breast tumor cell lines. Preliminary screening indicated that compound 7 g demonstrated significant growth inhibitory potential against all evaluated cell lines. The results of structure-activity relationship study exhibited that quinolone derivatives are superior in cytotoxic potential compared to 1, 8-naphthyridone series. Furthermore, ethyl quinolone derivatives were more potent cytotoxic agents comparing with cyclopropyl quinolones.

## Introduction

Quinolones are bicyclic ring structures containing 4-oxo-1, 4-dihydroquinoline core and different substituted moieties at N-1 position and demonstrate a wide range of pharmacological effects (1-3). Some important quinolone derivatives (*e.g*. ciprfloxacin) containing various substitutes at different positions of quinolone central core, have been primarily known as potent antibacterial agents that target type II bacterial topoisomerases (DNA gyrase and topoisomerase IV) and efficiently inhibit DNA replication and transcription by generating several double-stranded DNA break (4-6). Several researches have indicated that some quinolone derivatives display inhibitory potential against eukaryote topoismarase II. Therefore, in addition to antibacterial activity, some member of quinolones demonstrated substantial dose-dependent growth inhibitory potential against some cancerous cells (7-10). Moreover, a variety of cytotoxic mechanism such as apoptotic induction and inhibition of tubulin polymerization have been postulated as a plausible mechanism responsible for anticancer activity of cytotoxic quinolones (11-13). 

**Figure 1:**
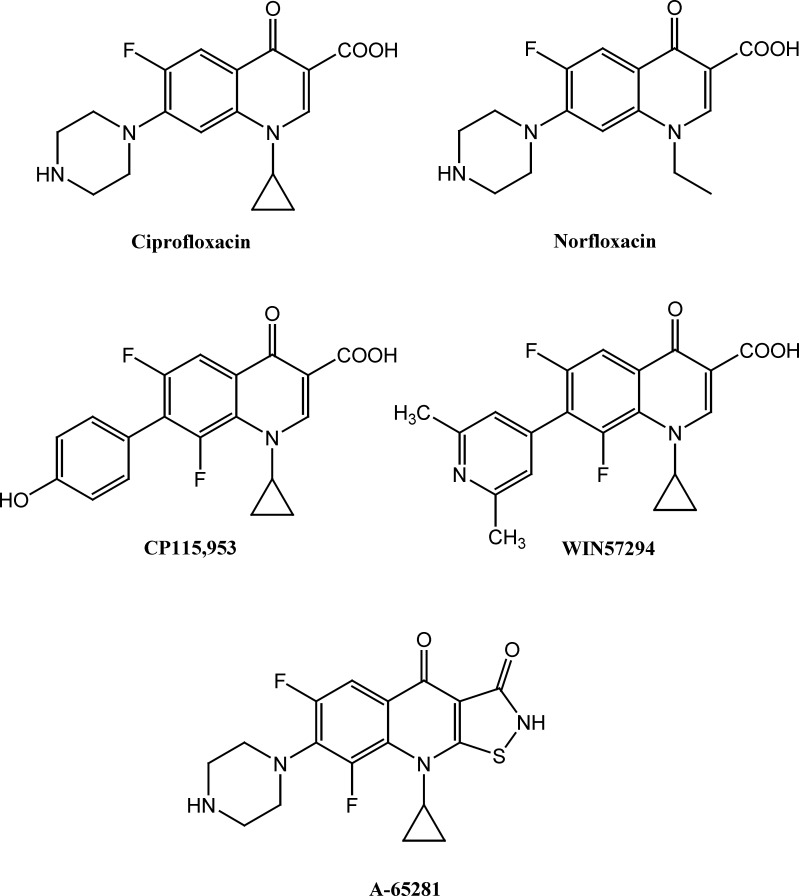
Chemical structure of antibacterial (Ciprofloxacin and Norfloxacin) and some anticancer quinolones (CP115,953, WIN57294 and A-65281

According to above findings and based on ciprofloxacin and norfloxacin core (Figure 1), several novel quinolone derivatives were synthesized and displayed significant anticancer activity. Structure-activity relationship study of cytotoxic quinolones demonstrated that substitution of aromatic moieties at C-7 position of quinolone central nucleus mainly affect the anticancer activity of these compounds and enhances selectivity toward type II of human topoisomerase rather than bacterial type (9, 14, 15). Chemical structure of some potent cytotoxic quinolones containing aromatic and heteroaromatic substitutes at C-7 position such as 7-pyridinyl (WIN57294) and 7-hydroxyphenyl (CP-115, 953) are demonstrated in Figure 1 (9). Moreover, 7-pyperzinyl derivatives of tricyclic quinolones such as isothiazoloquinolones A-65281 (Figure 1) induces significant DNA-breakage mediated by Calf thymus topoisomerase II and demonstrated cytotoxic potential nearly as potent as etoposide (16). We have previously reported cytotoxic activity of *N*-substituted pipearzinyl quinolone derivatives of ciprofloxacin type containing *N*-2-(furyl-2-yl)-2 (chlorobenzyloxyimino) ethyl moiety, *N*-[2-(5-chlorothiophen-2-yl)-2-oxoethyl] or *N*-[2-(5-chlorothiophen-2-yl)-2-oxyiminoethyl]piperazinyl moiety against some human tumor cell lines (17, 18). The results indicate that introduction of aforementioned substitutes into piperazine ring of quinolone derivatives resulted in enhanced cytotoxic potential in some cases. In continuation of our ongoing research program on finding the potent anticancer scaffolds (19-23) and in attempt to construct structure-activity relationship of these type of cytotoxic quinolones, in the current study, we report the synthesis and cytotoxic activity of novel derivatives of 6-fluoro-quinolone 3-carboxylic acid and 6-fluoro-1,8-naphthyridone 3-carboxylic acid core containing *N*-2-(2-furyl)-2-(chlorobenzyloxyimino) ethyl or *N*-2-(3-furyl)-2-(chlorobenzyloxyimino) ethyl piperazinyl pendant. 

## Experimental

*Chemistry*

A Kofler hot stage apparatus was used for the measurement of reported melting. The IR spectra were recorded on a Nicollet FT-IR Magna 550 spectrometer. The ^1^HNMR spectra were recorded on Bruker FT-500 MHz spectrometer and chemical shifts (δ) are reported in ppm relative to internal tetramethylsilane. Mass spectra were recorded on an Agilent Technology (HP) mass spectrometer operating at an ionisation potential of 70 eV. Analytical thin layer chromatography (TLC) on Merck silicagel 60 F254 plates using various mobile phases of different polarities was performed in order to confirm the purity of final products. 

*General method for the synthesis of O-benzyloximes derivatives 5a-f*

**Scheme 1.**
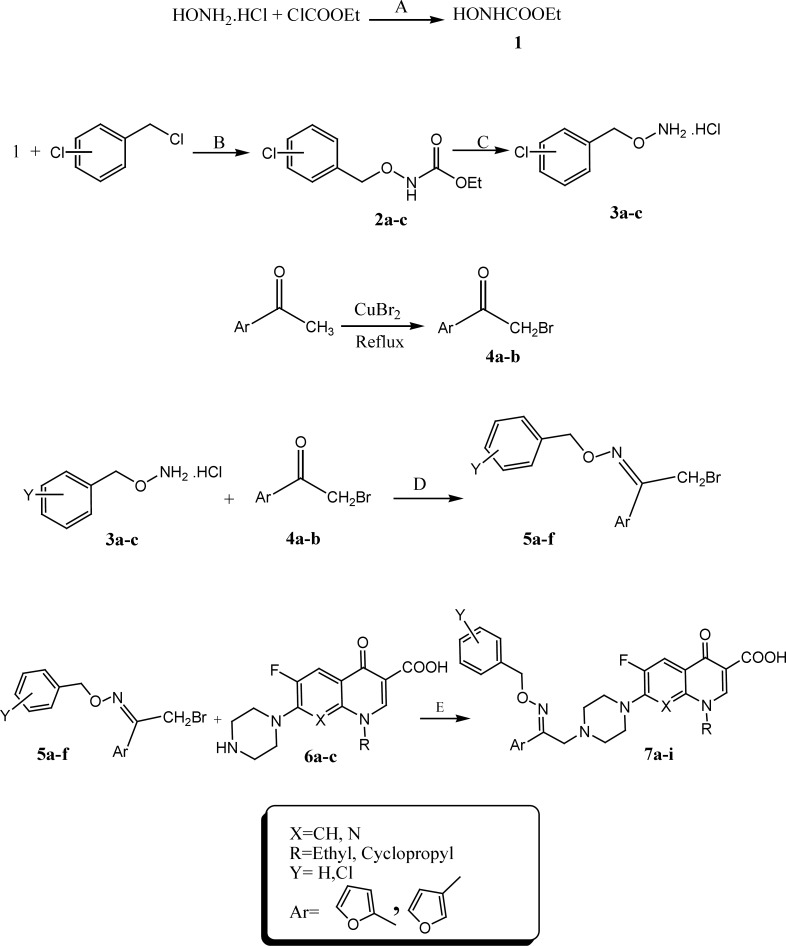
Synthesis of *N*-2-(furyl)-2-(chlorobenzyloxyimino)ethyl quinolones (**6a-i**). Reagents and conditions: A) Na_2_CO_3_, H_2_O, rt; B) Na, EtOH, rt; C) 1. NaOH, H_2_O, heat, 2. EtOH, HCl; D) MeOH, rt; E) DMF, NaHCO_3_, rt

To a mixture of hydroxylamine hydrochloride (0.4 mol), Sodium carbonate anyhydrous (0.6 mol) in 185 mL H_2_O was dropped ethyl chloro formate (0.23 mol). After completion of the reaction, precipitate was extracted with diethyl ether to give *N*-hydroxyurethane 1. Stirring a mixture of compound 1(0.68 mol) and chlorobenzylchloride derivatives (0.42 mol) in 120 mL EtOH gave chlorobenzyl carbethoxy hydroxamate derivatives 2a-c. Compound 2a-c (40 mmol) and sodium hydroxide (80 mmol) in 50 mL EtOH was refluxed for 2 hours. The precipitated product 3a-c was filtered off, dried, and recrystallized from ethanol and water. Compounds 5a-f were prepared from 2-bromo-(furan -2 or 3- yl)-ethanone 4a-b (1 mmol) and substituted *O*-benzylhydroxylamine hydrochlorides 3a-c (2 mmol) in methanol (10 mL) at room temperature for 3 days (Scheme 1) (19). 

*General method for the synthesis of N-2-(2 or 3-furyl)-2-(chlorobenzyloxyimino) ethyl quinolone (7a-i)*

A mixture of 2-bromo-1-(furan-2 or 3-yl) ethanone *O*-2-chlorobenzyl oxime derivatives 5a-f (0.55 mmol), quinolone derivatives 6a-c (0.5 mmol) and NaHCO_3_ (0.5 mmol) in DMF (5 mL) was stirred at room temperature for 6-9 days. After completion, water (20 mL) was added and the precipitate was filtered off, washed with water and recrystallized from EtOH-CHCl_3 _to give target compounds 7a-i.

*7-(4-(2-(benzyloxyimino)-2-(furan-3-yl) ethyl) piperazin-1-yl)-1-ethyl-6-fluoro-4-oxo-1, 4-dihydroquinoline-3-carboxylic acid* *7a *(18)

Yield 54%; m.p. 217 - 218C; ^1^HNMR (500 MHz, DMSO-d_6_): 1.40 (t, 3H, *J*=7 Hz, CH_3_), 3.23 -3.32 (m, 4H, piperazine), 3.58-3.65 (m, 4H, piperazine), 3.62 (s, 2H, CH_2_), 4.58 (q, 2H, *J*=7 Hz, CH_2_-CH_3_), 5.20 (s, 2H, OCH_2_), 6.71 (s, 1H, furyl), 7.17 (d, 1H, *J*=7.1Hz, H_8_), 7.29-7.43 (m, 5H, phenyl), 7.70 (s, 1H, furyl), 7.93 (d, 1H, *J*=13.2Hz, H_5_), 8.25 (s, 1H, furyl), 8.95 (s, 1H, H_2_), 15.34 ppm (s, 1H, COOH). IR (KBr): 1681, 1619 (C=O). MS *m/z* 532 (M^+^, 5), 425 (12), 332 (100), 139 (26).

*7-(4-(2-(4-chlorobenzyloxyimino)-2-(furan-2-yl) ethyl) piperazin-1-yl)-1-ethyl-6-fluoro-4-oxo-1, 4-dihydroquinoline-3-carboxylic acid* *7b*

Yield 85%; m.p. 248–250C; ^1^HNMR (500 MHz, DMSO-d_6_): 1.40 (t, *J*=7 Hz, 3H, CH_3_), 2.62-2.70 (m, 4H, piperazine), 3.23-3.30 (m, 4H, piperazine), 3.64 (s, 2H, CH_2_), 4.57 (q, *J*=7.0 Hz, 2H, CH_2_), 5.20 (s, 2H, OCH_2_), 6.56-6.70 (m, *J*=3.3 Hz, 1H, furyl), 7.15 (d, *J*=7.15 Hz, 1H, H_8_), 7.43-7.49 (m, 4H, benzyl), 7.74 (s,1H, furyl), 7.92 (d, *J*=13.2 Hz, 1H, H_5_), 8.95 (s,1H, H_2_), 15.14 ppm (s, 1H, COOH); IR (KBr): 1731, 1629 (C=O). MS *m/z* 566 (M^+^, 3), 332 (40), 316 (21), 233 (25), 125 (100).

*7-(4-(2-(2-chlorobenzyloxyimino)-2-(furan-2-yl) ethyl) piperazin-1-yl)-1-ethyl-6-fluoro-4-oxo-1, 4-dihydroquinoline-3-carboxylic acid 7c*

Yield 73%; m.p. 277–278C; ^1^HNMR (500 MHz, DMSO-d_6_): 1.40 (t, *J*=6.8 Hz, 3H, CH_3_), 2.45-2.76 (m, 4H, piperazine), 3.25-3.53 (m, 4H, piperazine), 3.61 (s, 2H, CH_2_), 4.56 (q, *J*=6.8Hz, 2H, CH_2_), 5.27 (s, 2H, OCH_2_), 6.50 (d,* J*=3.2 Hz, 1H, furyl), 6.98 (d, *J*=3.2 Hz, 1H, furyl), 7.14 (d, *J*=6.8 Hz, 1H, H_8_), 7.30-7.40 (m, 4H, benzyl), 7.55 (t, *J*=3.2 Hz, 1H, furyl), 7.89 (d, *J*=13.2 Hz, 1H, H_5_), 15.29 ppm (s, 1H, COOH); IR (KBr): 1741, 1629 (C=O). MS *m/z* 566 (M^+^, 2), 318 (19), 234 (24), 141 (100).

*7-(4-(2-(2-chlorobenzyloxyimino)-2-(furan-2-yl) ethyl) piperazin-1-yl)-1-ethyl-6-fluoro-4-oxo-1, 4-dihydro-1, 8-naphthyridine-3-carboxylic acid 7d*

Yield 57%; m.p. 265–267C;^ 1^HNMR (500 MHz, DMSO-d_6_): 1.38 (t, *J*=6.8Hz, 3H, CH_3_), 2.80-2.91 (m, 4H, piperazine), 3.62 (s, 2H, CH_2_), 3.73-3.86 (m, 4H, piperazine), 4.46 (q,* J*=6.8 Hz, 2H, CH_2_), 5.26 (s, 2H, OCH_2_), 6.58(d, *J *= 3.2 Hz, 1H, furyl), 6.99 (d, *J*= 3.2Hz, 1H, furyl), 7.28-7.59 (m, 4H, benzyl), 8.02 (d, *J *= 13.6 Hz, 1H, H_5_), 8.06 (bs, 1H, furyl), 8.92 (s, 1H, H_2_), 15.15 ppm (s, 1H, COOH); IR (KBr): 1629 (C=O). MS *m/z* 566 (M^+^, 2), 319 (18), 234 (21), 141 (100).

*7-(4-(2-(4-chlorobenzyloxyimino)-2-(furan-3-yl) ethyl) piperazin-1-yl)-1-ethyl-6-fluoro-4-oxo-1, 4-dihydroquinoline-3-carboxylic acid 7e*

Yield: 48%; m.p. 270–272 C; ^1^HNMR (500 MHz, DMSO-d_6_): 1.41(t, *J*=7.0 Hz, 3H, CH_3_), 2.56-2.64 (m, 4H, piperazine), 3.20-3.28 (m, 4H, piperazine), 3.61(q, *J*=7.0 Hz, 2H, CH_2_), 5.15 (s, 2H, OCH_2_), 6.69 (d, *J*=2.7 Hz, 1H, furyl), 6.95 (d, *J*=2.7 Hz, 1H, furyl), 7.15 (d, *J*=7.1 Hz, 1H, H_8_), 7.42-7.46 (m, 4H, benzyl), 7.73 (bs, 1H, furyl), 7.90 (d, *J*=13.2 Hz, 1H, H_5_), 8.93 (s, 1H, H_2_), 15.12 ppm (s, 1H, COOH); IR (KBr): 1728, 1631 (C=O). MS *m/z* 566 (M^+^, 2), 318 (31), 234 (17), 141 (100).

*7-(4-(2-(4-chlorobenzyloxyimino)-2-(furan-3-yl) ethyl) piperazin-1-yl)-1-ethyl-6-fluoro-4-oxo-1,4-dihydro-1,8-naphthyridine-3-carboxylic acid 7f*

Yield: 66%; m.p. 230-231 C; ^1^HNMR (500 MHz, DMSO-d_6_): 1.36 (t, *J*=6.7 Hz, 3H, CH_3_), 2.53-2.59 (m,4H, piperazine), 3.57 (s, 2H, CH_2_), 3.70-3.80 (m, 4H, piperazine), 4.46 (q, *J*=6.7 Hz, 2H, CH_2_), 5.13 (s, 2H, OCH_2_), 6.68 (bs, 1H, furyl), 6.95 (bs, 1H, furtyl), 7.68-7.86 (m, 4H, benzyl), 7.74 (bs, 1H, furyl), 8.06 (d, *J*=13.5Hz, 1H, H_5_), 8.94 (s, 1H, H_2_), 15.14 ppm (s, 1H, COOH); IR (KBr): 1720, 1634 (C=O). MS *m/z* 566 (M^+^, 6), 319 (14), 234 (33), 141 (100).

*7-(4-(2-(3-chlorobenzyloxyimino)-2-(furan-3-yl)ethyl)piperazin-1-yl)-1-ethyl-6-fluoro-4-oxo-1,4-dihydroquinoline-3-carboxylic acid 7g*

Yield: 49%; m.p. 229–230 C; ^1^HNMR (500 MHz, DMSO-d_6_):1.40 (t, *J*=6.3 Hz, 3H, CH_3_), 2.56-2.64 (m, 4H, piperazine), 3.22-3.30 (m, 4H, piperazine), 3.63 (s, 2H, CH_2_), 4.56 (q, *J*=6.3 Hz, 2H, CH_2_), 5.18 (s, 2H, OCH_2_), 6.68 (bs, 1H, furyl), 6.96 (bs, 1H, furyl), 7.15 (d,* J*=7.1Hz, 1H, H_8_), 7.34-7.42 (m, 3H, benzyl), 7.74 (bs, 1H, furyl), 7.88 (s, *J*=13.2 Hz, 1H, H_5_), 8.48 (s, 1H, benzyl), 8.93 (s, 1H, H_2_), 15.30 ppm (s, 1H, COOH); IR (KBr): 1725, 1626 (C=O). MS *m/z* 566 (M^+^, 6), 318 (29), 234 (16), 141 (100).

*7-(4-(2-(3-chlorobenzyloxyimino)-2-(furan-3-yl)ethyl)piperazin-1-yl)-1-cyclopropyl-6-fluoro-4-oxo-1,4-dihydroquinoline-3-carboxylic acid 7h*

Yield: 65%; m.p. 269–270 C; ^1^HNMR (500 MHz, DMSO-d_6_): 1.16-1.19 (m, 2H, cyclopropyl), 1.29-13.33 (m, 2H, cylopropyl), 2.50-2.52 (m, 4H, piperazine), 2.98 (s, 2H, CH_2_), 3.28-3.34 (m,4H, piperazine), 3.82-3.89 (m, 1H, cyclopropyl), 5.22 (s, 2H, OCH_2_), 6.63-6.65 (m, 1H, furyl), 6.89-6.98 (m, 1H, furyl), 7.31-7.41 (m, 3H, benzyl), 7.55 (d,* J*=6.9 Hz, 1H, H_8_), 7.76-7.83 (m,1H, furyl), 7.90 (d, *J*=13.1Hz, 1H, H_5_), 8.67 (s, 1H, H_2_), 15.13 ppm (s, 1H, COOH); IR (KBr): 1712, 1624 (C=O). MS *m/z* 578 (M^+^, 2), 344 (34), 246 (7), 141 (100). 

*7-(4-(2-(3-chlorobenzyloxyimino)-2-(furan-3-yl)ethyl)piperazin-1-yl)-1-ethyl-6-fluoro-4-oxo-1,4-dihydro-1,8-naphthyridine-3-carboxylic acid 7i*

Yield: 35%; m.p. 278–280 C; ^1^HNMR (500 MHz, DMSO-d_6_): 1.37 (t, *J*=6.9 Hz, 3H, CH_3_), 2.48-2.59 (m, 4H, piperazine), 3.33 (s, 2H, CH_2_), 3.74-3.80 (m, 4H, piperazine), 4.46 (q, *J*=6.9 Hz, 2H, CH_2_), 5.22 (s, 2H, OCH_2_), 6.95 (d, *J*=1.5Hz, 1H, furyl), 6.97(d, *J*=1.5 Hz, 1H, furyl), 7.31-7.45 (m, 3H, benzyl), 7.75 (t, *J*=1.5 Hz, 1H, furyl), 8.04 (d, *J*=13.5 Hz, 1H, H_5_), 8.49 (s, 1H, benzyl), 8.95 (s, 1H, H_2_), 15.30 ppm (s, 1H, COOH); IR (KBr): 1719, 1634 (C=O). MS *m/z* 567 (M^+^, 3), 319 (19), 234 (41), 141 (100).

*Cytotoxic evaluation*

The synthesized compounds 7a-i were tested against different human breast tumor cell lines including MCF-7, MDA-MB-231 and T47D using MTT (3-(4, 5-dimethylthiazol-2-yl)-2,5-diphenyl tetrazolium bromide) reduction assay. The cell lines were purchased from National Cell Bank of Iran (NCBI). Cells were seeded in 96-well plates at the density of 10,000 viable cells per well and incubated at 37º C in a humidified atmosphere with 5% CO_2_ for 24 h to allow cell attachment. The cells were then incubated for another 48 h with various concentrations of compounds 7a-i. The synthetic compounds were dissolved in DMSO and the final concentration of DMSO in each well was kept below 1%. etoposide was used as a positive control for each cell line. The medium was replaced with 200 µL RPMI-1640 without phenol red containing 0.5 mg/mL MTT. An additional 4h of incubation at 37 °C were done and then the medium was discarded. Dimethyl sulfoxide (100 μL) was added to each well and the solution was vigorously mixed to dissolve the purple tetrazolium crystals. The absorbance of each well was measured by plate reader (Biotek Instruments, Winooski, Vt.) at a test wavelength of 492 nm. Three independent experiments in triplicate were performed for determination of sensitivity to each compound. The IC_50_ were calculated by linear regression analysis, expressed in mean ± SD.

## Results and Discussion

*Chemistry*

The target compounds 7a-i were synthesized according to the procedure depicted in Scheme 1. Bromination of 1-(furan-2 or 3)-yl)ethanone in the presence of cupper II bromide in refluxing chloroform-ethyl acetate led to the formation of 1-(furan-2 or 3)-yl)ethanone 4a-b. Reaction of hydroxylamine hydrochloride and ethylchloroformate in sodium carbonate resulted in synthesis of ethyl hydroxy carbonate 1. Chlorobenzyl carbetoxy hydroxamate derivatives 2a-c were synthesized through the reaction of ethyl hydroxy carbonate 1 and different chlorobenzyl chloride derivatives in presence of sodium ethanolate at room temperature. Hydrolysis of resulted compounds 2a-c under basic condition led to production of *O*-chlorobenzylhydroxylamine hydrochloride 3a-c. Reaction of compounds 3a-c and 2-bromo-1-(furan-2 or 3)-yl) ethanone 4a-c in methanol resulted in synthesis of 2-bromo-1-(furan-2 or 3)-yl)ethanone *O*-chlorobenzyl oxime derivatives 5a-f. *N*-2-(2 or 3-furyl)-2-(chlorobenzyloxyimino) ethyl quinolone derivatives 7a-i were synthesized by reaction of different quinolone derivatives 6a-c and corresponding 2-bromo-1-(furan-2 or 3-yl)ethanone *O*-chlorobenzyloxime 5a-f in DMF in the presence of NaHCO_3_ at ambient temperature (Scheme 1). Chemical structures of target compounds were characterized by IR, ^1^HNMR and elemental analysis.

*Cytotoxic activity*

The cytotoxic activity of target compounds 7a-i were assessed *in-vitro* against a panel of three human cancer cell lines including MCF-7, MDA-MB-231 and T47D. The percentage of cell growth was evaluated using MTT colorimetric assay in comparison with untreated controls. For each compound, the 50% inhibitory concentration (IC_50_) was determined and reported in Table 1. The corresponding data for etoposide was included for comparison. On the basis of obtained IC_50_ values, most of synthesized compounds demonstrated potent to moderate cytotoxic potential especially against T47D cell line ((IC_50_=2.20-39.08μM). However; most of evaluated derivatives were almost inactive against MDA-MB-231 cell line (IC_50_>100 μM). The most promising compound of this series 7g, ethyl quinolone derivative (X=CH and R=Et) containing 3-furyl moiety (Ar=3-furyl) and 3-chloro phenyl (Y=3-Cl), demonstrated significant growth inhibitory potential against all evaluated cell lines (the corresponding IC_50_ values against MCF-7, MDA-MB-231 and T47D cells were 3.03, 11.90 and 2.20 μM, respectively). Moreover, 7 g demonstrated superior cytotoxic activity compared to reference compound, etoposide (the corresponding IC_50_ values against MCF-7, MDA-MB-231 and T47D cells were 7.90.5, 11.11.1 and 80.8 μM, respectively).

Considering the X-substituted group on the central core, synthesized compounds could be categorized into two groups: quinolone and 1,8-naphthyridinones containing CH and N group in place of X-substitute, respectively. According to obtained cytotoxic data (Table 1), the following structure-activity relationship might be developed:

- Quinolone derivatives were more potent cytotoxic agents than 1, 8-naphthyridones in most cases; e.g ethyl quinolone compound 7 g (Ar=3-furyl, Y=3-Cl) was found to be most potent cytotoxic derivative. However, its 1-ethyl 1,8-naphthyridinone counterpart 7i was almost inactive against MCF-7 and MDA-MB-231 (IC_50_>100μM) and showed weak growth inhibitory potential toward T47D cells (IC_50_=39.08μM).
- Ethyl quinolones demonstrated high cytotoxic potential over cyclopropyl quinolones; *e.g*. compound 7 g was potent cytotoxic agent of these series (the corresponding IC_50_ values against MCF-7, MDA-MB-231 and T47D cells were 3.03, 11.90 and 2.20 μM, respectively). While its cyclopropyl quinolone counterpart 7h was inactive against MCF-7 and MDA-MB-231 cancer cells (IC_50_>100 μM) and showed moderate cytotoxic potential against T47D cells (IC_50_=12.35μM).
- Considering the substituted moiety on the oxime pendant (Ar group), no significant differences were observed between cytotoxic potentials of 2-furyl and 3-furyl derivatives; *i.e*. compound 7e, ethyl quinolone derivative containing 3-furyl substitute on the oxime pendant (Y=4-Cl) and its 2-furyl counterpart 7b, demonstrated potent cytotoxic potential against MCF-7 (7b (IC_50_=12.35 μM) and 7e (IC_50_=11.40 μM)) and T47D cells (7b (IC_50_=8.70μM) and 7e (IC_50_=10.90μM)). Both compounds were almost inactive against MDA-MB-231 cells.

**Table 1:** Structures and *in-vitro* cytotoxicity of compounds 7a-i against three different human breast cancer cell lines assessed by MTT reduction assay^a^.

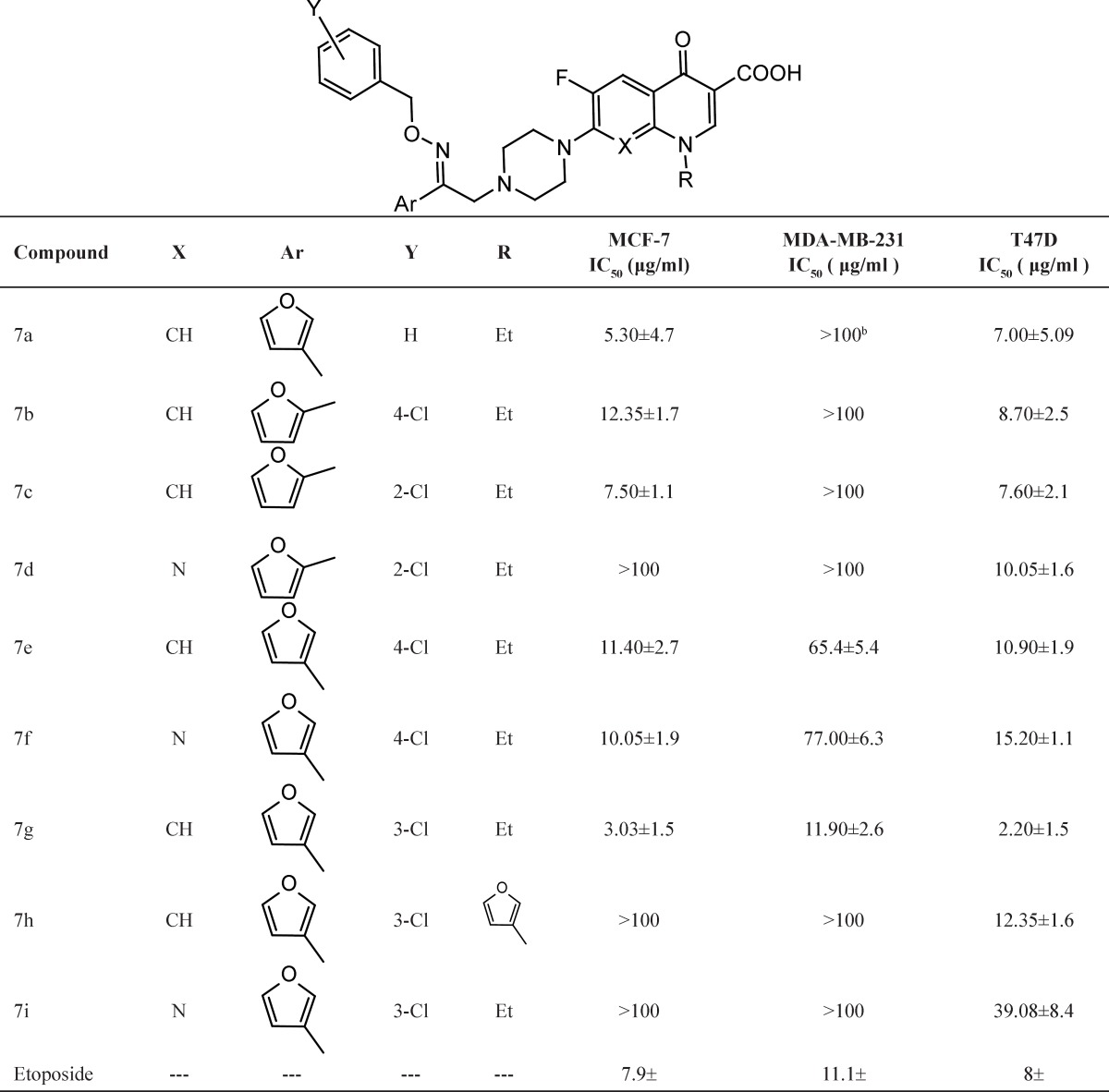

a Values represent the meanSD of three different experiments.

b Compounds were tested at the maximum final concentration of 100 μg/mL.

## Conclusion

In conclusion, a novel series of 6-fluoro quinolone 3-carboxylic acid and 6-fluroro-1,8-naphthyridone 3-carboxylic acid derivatives containing *N*-2-(2-furyl)-2-(chlorobenzyloxyimino)ethyl or *N*-2-(3-furyl)-2-(chlorobenzyloxyimino)ethyl piperazinyl pendant attached to the central core was synthesized and evaluated against three different human cancer cell lines. The most promising compound of ethyl quinolone series, 7 g, demonstrated significant growth inhibitory potential against all evaluated cell lines. The results of structure-activity relationship study demonstrated that quinolone derivatives are superior in cytotoxic potential compared to 1,8-naphthyridone series. Furthermore, ethyl quinolone derivatives were more potent cytotoxic agents than cyclopropyl quinolones. 
